# Implementation and Outcomes of an Early Rehabilitation Program in a Tertiary Emergency and Critical Care Center: A Single-Center Historical Cohort Study

**DOI:** 10.3390/life16040587

**Published:** 2026-04-01

**Authors:** Hidetaka Onda, Shoji Yokobori, Masato Miyauchi

**Affiliations:** 1 Department of Disaster and Emergency Medicine, Kochi University, Kochi 783-8505, Japan; 2 Department of Advanced Emergency and Critical Care Center, Nippon Medical School Hospital, Tokyo 113-8603, Japan

**Keywords:** intensive care unit, early mobilization, ICU-acquired weakness, post-intensive care syndrome, propensity score, overlap weighting, discharge disposition

## Abstract

Early rehabilitation may reduce the likelihood of long-term disability after critical illness, but in high-turnover emergency and critical care settings, benefits other than improved survival may appear at discharge. This retrospective historical cohort study assessed the impact of a standardized rehabilitation system initiated within 24 h of hospital arrival on inpatient and discharge outcomes. The study included consecutive intensive care unit admissions between 2011 and 2023 and compared periods before and after 1 April 2022 when the system was introduced. Outcomes included in-hospital mortality, frequency of a favorable discharge outcome (Glasgow Outcome Scale score indicating good recovery or moderate disability), rate of transfer to a rehabilitation hospital, and length of hospital stay. Between-period differences were adjusted using propensity-score overlap weighting. Among 20,252 patients, adjusted analyses showed no significant differences in in-hospital mortality (odds ratio [OR] 1.17, 95% confidence interval [CI] 0.81–1.69) or length of stay (mean difference −0.12 days, 95% CI −1.28 to 1.04). After implementation, there was a likelihood of a favorable discharge outcome (OR 1.74, 95% CI 1.29–2.36) and transfer to a rehabilitation hospital (OR 1.77, 95% CI 1.23–2.54). This change was associated with a more favorable transition to post-acute rehabilitation without improving short-term mortality.

## 1. Introduction

Although advances in intensive care have steadily improved the patient survival rate, a substantial proportion of survivors continue to experience persistent impairments after discharge, and intensive care unit (ICU)-acquired weakness and post-intensive care syndrome are now widely recognized as major clinical challenges [1,2]. ICU-acquired weakness arises from the interplay of multiple factors, including systemic inflammation, prolonged bed rest and immobility, and neuromuscular dysfunction, and is known to compromise activities of daily living and to impair quality of life well into the post-acute phase [1]. Furthermore, there have been reports of long-lasting cognitive decline and psychological sequelae after an ICU admission [3,4,5], and delirium in particular has been established as an independent risk factor for cognitive deterioration [6,7]. These impairments affect not only patients but also their families and society at large and are associated with rehospitalization and delayed return to social functioning [2,3,4,5].

To address these problems, comprehensive rehabilitation centered on early mobilization has been recommended alongside optimization of analgesia, sedation, and management of delirium [8,9] and systematized as the ICU liberation bundle (also known as the ABCDEF bundle) [10]. In multicenter implementation studies, improved adherence to this bundle has been accompanied by better outcomes across several domains, including discharge destination and delirium [11]. Moreover, early activity in the ICU has been shown to be safe and feasible in both observational studies and randomized controlled trials [12,13]. Recent observational data in neurocritical care have also suggested that initiating rehabilitation early during intensive care may be associated with improved outcomes [14]. However, the magnitude of benefit from early rehabilitation is not consistent, appearing to depend heavily on the context of implementation, including how early the intervention is initiated, its intensity, severity of illness, the baseline level, and degree of standardization of usual care (including sedation and mechanical ventilation practices), and baseline mobilization levels in the comparison group [15,16,17,18,19,20]. Even in recent large-scale trials, aggressive early mobilization has not necessarily improved primary outcomes [20], highlighting the fact that evaluation of early rehabilitation requires a contextual interpretation grounded in both the content of the intervention and the conditions under which it can be delivered.

In this regard, Japanese tertiary emergency and critical care centers constitute a distinctive operational context. Under systems predicated on short lengths of stay and high patient turnover, these centers must simultaneously deliver resuscitation, intensive care, postoperative management, and coordination of interfacility transfer, which imposes structural constraints on achieving substantial functional recovery during hospitalization. Accordingly, the significance of early rehabilitation in this setting lies less in producing marked short-term functional gains than in mitigating the trajectory of deconditioning and accelerating the initial steps of mobilization and resumption of activity, thereby facilitating a smooth transition to post-acute rehabilitation. In such a context, evaluation should incorporate not only in-hospital endpoints such as mortality but also practical outcomes that connect to the potential for recovery after discharge, including status at discharge and discharge destination [21]. Therefore, we performed this study to determine how implementation of an acute-phase rehabilitation system initiated within 24 h of hospital arrival in a tertiary emergency and critical care center influences routinely available inpatient clinical indicators and practical discharge-related outcomes using real-world clinical data.

## 2. Objective

The objective of this study was to determine whether implementation of an acute-phase rehabilitation program initiated within 24 h of hospital arrival in a tertiary emergency and critical care center can be appropriately evaluated within a framework that uses routinely and universally obtainable inpatient clinical indicators and practical outcomes related to decision-making regarding discharge destination. Under a standardized operational definition, we implemented acute-phase rehabilitation and prespecified an analysis plan and measurement items to ensure comparability with routine clinical care during the non-intervention period.

## 3. Methods

### 3.1. Study Design and Setting

This study had a single-center, retrospective historical cohort design and analyzed data collected for admissions to our ICU between 1 January 2011 and 31 December 2023. Using the implementation date of the acute-phase rehabilitation system (1 April 2022) as the boundary, patients were divided into a non-intervention period (1 January 2011 to 31 March 2022) and an intervention period (1 April 2022 to 31 December 2023).

### 3.2. Participants

Consecutive patients admitted to the emergency and critical care center and subsequently to the ICU during the study period were included. Patients who were in cardiac arrest at the time of hospital admission were excluded. For patients with more than one admission during the study period, only the first admission was included.

### 3.3. Definitions of Intervention and Non-Intervention

Hospital arrival was defined as the time of presentation to our center and corresponds to the hospital admission time entered in the electronic medical record. The intervention was defined as the system-wide implementation, without exception for any ICU admission, of a practice whereby acute-phase rehabilitation was initiated within 24 h of hospital arrival. Considering that all patients in this study were admitted to the ICU soon after hospital arrival, the ICU admission date was used as the index date for cohort definition and follow-up, whereas the intervention itself was operationalized as initiation of rehabilitation within 24 h of hospital arrival. In this study, the intervention (exposure) did not refer to rehabilitation as an isolated technique; rather, it was defined as implementation of a multidisciplinary operational design and standardization that enables early intervention (a rehabilitation-ready ICU system). Rehabilitation was led primarily by physical therapists and was performed for approximately 1 h/day. Given that ICU procedures, including analgesia and sedation, management of mechanical ventilation, and management of delirium, may be interdependent with the feasibility of early intervention, the estimated associations should be interpreted as findings related to a change in the bundled practice accompanying system implementation. After implementation, even under sedation or mechanical ventilation, the operational policy was to initiate care with passive interventions, such as range-of-motion exercises, positioning, postural changes, and thoracic mobilization, within the range in which safety could be ensured. When sedation was lightened according to the patient’s condition and discussion with the attending physician, more active range-of-motion training was carried out. Because emergency and critical care physicians were available on site 24 h a day, physical therapists could discuss the patient’s condition with the attending physician at any time, and rehabilitation progressed stepwise to sitting at the edge of the bed, standing, and ambulation when judged feasible. In parallel, speech-language therapists, together with the swallowing team, assessed readiness for oral intake and supported progression to oral feeding, particularly after extubation. Thus, the intervention was implemented as a standardized operational workflow tailored to the patient’s condition rather than as a rigid protocol with fixed progression criteria for every patient. For analysis, the intervention group comprised patients admitted to the ICU on or after the implementation date and the non-intervention group consisted of those admitted before the implementation date.

### 3.4. Outcomes

The primary outcomes were (1) in-hospital mortality, (2) a favorable discharge outcome, and (3) transfer to a post-acute rehabilitation hospital. Although the Glasgow Outcome Scale (GOS) was originally developed for patients with brain injury [22], it was used in this study as a global outcome measure that is routinely available for all patients across heterogeneous ICU populations. More granular functional scales, such as the modified Rankin Scale and Functional Independence Measure, were not systematically obtained for all patients; therefore, the GOS was used as a facility-wide measure suitable for evaluation purposes. Transfer to a post-acute rehabilitation hospital was positioned not as a surrogate for functional improvement per se, but as a process-of-care outcome reflecting linkage between acute care and post-acute rehabilitation. Given that transfer can be influenced by regional bed availability, referral pathways, and coordination processes, it was interpreted as an indicator of the transition process that connects patients with a post-discharge recovery trajectory. Length of hospital stay was treated as an exploratory outcome.

### 3.5. Covariates

The following prespecified covariates were used for propensity score estimation: age, sex, the Glasgow Coma Scale (GCS) score (an indicator of consciousness level at presentation), systolic and diastolic blood pressure (BP), heart rate, respiratory rate, body temperature, blood gas variables (pH, arterial partial pressure of carbon dioxide, arterial oxygen saturation), the Acute Physiology and Chronic Health Evaluation II (APACHE II) score [23], and results of early laboratory tests (lactate, sodium, creatine kinase, aspartate aminotransferase, alanine aminotransferase, amylase, blood urea nitrogen, creatinine, albumin, C-reactive protein, and D-dimer).

### 3.6. Handling of Missing Data

Missing covariate values were imputed using multiple imputation by chained equations (m = 20). Predictive mean matching was used for continuous variables, logistic regression for binary variables, and ordinal logistic regression for ordinal variables.

### 3.7. Propensity Score and Weighting

Propensity scores were estimated using logistic regression with the intervention indicator as the dependent variable and the prespecified covariates as independent variables. Overlap weights were applied (intervention group, 1—propensity score; non-intervention group, propensity score).

### 3.8. Statistical Analysis

Unadjusted between-group comparisons were performed using Fisher’s exact test for categorical variables and the Wilcoxon rank-sum test for continuous variables. Covariates with missing values were imputed using multiple imputation by chained equations (m = 20), and the imputation model included the intervention indicator and primary outcomes. The same analytic procedure was applied for each imputed dataset: (1) propensity scores were estimated by logistic regression using the prespecified covariates; (2) overlap weights were assigned (intervention group, 1—propensity score; non-intervention group, propensity score); and (3) outcomes were estimated using weighted regression models (weighted logistic regression for binary outcomes and weighted linear regression for length of hospital stay). Robust variances were calculated for the weighted regression models using sandwich estimators. Estimates and variances from each imputed dataset were pooled using Rubin’s rules. Covariate balance was evaluated using the SMD, with an absolute value of <0.10 considered a practical target. All statistical analyses were performed using R software 4.5.2 (R Foundation for Statistical Computing, Vienna, Austria). A two-sided *p*-value of <0.05 was considered statistically significant. An exploratory analysis stratified by disease category was also performed. Disease classification was defined based on the principal diagnosis at admission, and the results are presented in (Supplementary Table S1).

### 3.9. Ethics Statement

This study was approved by the Ethics Committee of Nippon Medical School (approval number B-2023-654) and was conducted in accordance with the principles of the Declaration of Helsinki. Use of clinical information for research was conducted under an opt-out approach via in-hospital notices and the hospital website, and personally identifiable information was protected through linkage-capable anonymization. Because this was a retrospective study within the scope of secondary use of clinical information, the requirement for individual written informed consent was waived.

## 4. Results

### 4.1. Study Population

In total, 20,252 consecutive patients were admitted to the ICU during the study period, with 18,068 in the non-intervention period and 2184 in the intervention period.

### 4.2. Missing Data

Missingness in key covariates (non-intervention/intervention) was as follows: age 0.02% (5/0), body temperature 6.0% (1091/130), systolic BP 19.9% (3645/389), diastolic BP 19.6% (3572/392), heart rate 18.2% (3388/290), and respiratory rate 18.5% (3303/446). Missingness in outcomes was 0.005% for in-hospital mortality (1/0), 0.01% for the GOS score (2/0), and 0.5% for length of hospital stay (90/9). There were very few cases with missing outcomes; these were excluded from the corresponding outcome analyses, and multiple imputation was applied to covariates only.

### 4.3. Patient Characteristics and Early Clinical and Laboratory Findings

The median age was 67.0 years [interquartile range (IQR) 47, 79] in the non-intervention period and 68.0 years [IQR 48, 80] in the intervention period; the proportion of male patients was 62.0% and 60.1%, respectively. A marked between-group imbalance was observed in the median GCS score, which was 14 [IQR 6, 15] in the non-intervention period and 12 [IQR 3, 15] in the intervention period. Median body temperature was 36.5 °C [IQR 36.0, 37.1] in the non-intervention period and 36.7 °C [IQR 36.2, 37.3] in the intervention period. The respiratory rate also tended to be higher in the intervention period (22 [IQR 18, 30] vs. 21 [IQR 18, 27]/min); median arterial partial pressure of carbon dioxide was 35.8 mmHg [IQR 31.0, 41.4] in the intervention period and 38.2 mmHg [IQR 33.4, 44.6] in the non-intervention period, and only small-to-moderate imbalances were detected in pH and arterial oxygen saturation. The median D-dimer level was 3.7 mg/L [IQR 1.1, 15.6] in the non-intervention period and 1.9 mg/L [IQR 1.0, 7.9] in the intervention period; several laboratory variables, including lactate, albumin, AST, ALT, and D-dimer, showed absolute SMDs of ≥0.10 before weighting. Among available cases, the median APACHE II score was 18 [IQR 12, 27] in the non-intervention period and 15 [IQR 13, 24] in the intervention period (Table 1). Overall, the intervention cohort differed from the non-intervention cohort in terms of case mix, particularly with respect to consciousness level and selected physiological indicators.

### 4.4. Unadjusted Outcomes

In-hospital mortality was significantly lower in the intervention period than in the non-intervention period (306/2184 [14.0%] vs. 3650/18,068 [20.2%], *p* < 0.001). A favorable discharge outcome was significantly more common in the intervention period (1782/2184 [81.6%] vs. 12,250/18,068 [67.8%], *p* < 0.001), as was the rate of transfer to a post-acute rehabilitation hospital (269/2184 [12.3%] vs. 1102/18,068 [6.1%], *p* < 0.001). The median length of hospital stay was significantly longer in the intervention period (7.0 days [IQR 2, 16] vs. 6.0 days [IQR 2, 16], *p* < 0.001) (Table 2).

### 4.5. Propensity Score Overlap Weighting and Covariate Balance

**Figure 1:**
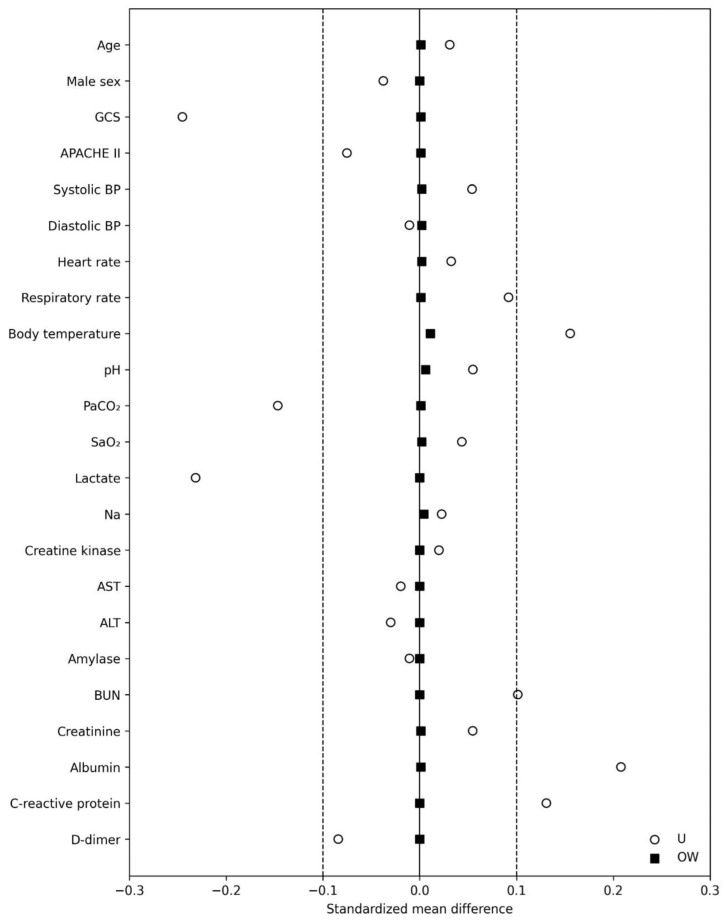
Love plot (before and after overlap weighting). Love plot of standardized mean differences before and after overlap weighting. Love plot showing standardized mean differences (SMDs) for baseline covariates between the non-intervention and intervention groups before and after overlap weighting. The solid vertical line indicates SMD = 0, and the dashed vertical lines indicate the prespecified balance threshold (|SMD| = 0.10). Values closer to zero after overlap weighting indicate improved covariate balance. Abbreviations: APACHE II, Acute Physiology and Chronic Health Evaluation II; ALT, alanine aminotransferase; AST, aspartate aminotransferase; BP, blood pressure; BUN, blood urea nitrogen; GCS, Glasgow Coma Scale; OW, overlap weighting; PaCO_2_, arterial partial pressure of carbon dioxide; SaO_2_, arterial oxygen saturation; SMD, standardized mean difference; U, unweighted.

Propensity scores were estimated using age, sex, GCS, systolic and diastolic BP, heart rate, respiratory rate, body temperature, blood gas variables (pH, arterial partial pressure of carbon dioxide, arterial oxygen saturation), APACHE II score, and early laboratory test results (lactate, sodium, creatine kinase, aspartate aminotransferase, alanine aminotransferase, amylase, blood urea nitrogen, creatinine, albumin, C-reactive protein, and D-dimer), and overlap weighting was applied. SMDs were calculated within each imputed dataset and summarized as representative values. Before weighting, the maximum absolute SMD was 0.291; after weighting, all variables had an absolute SMD of <0.10, with the maximum decreasing to 0.006, indicating improved covariate balance (Figure 1; Supplementary Table S2).

### 4.6. Overlap-Weighted Adjusted Effect Estimates

In the overlap-weighted analysis, there was no statistically significant difference in in-hospital mortality after implementation of the intervention (odds ratio [OR] 1.17, 95% confidence interval [CI] 0.81–1.69, *p* = 0.396). A favorable discharge outcome was significantly more common in the intervention period (OR 1.74, 95% CI 1.29–2.36, *p* < 0.001), as was the likelihood of transfer to a post-acute rehabilitation hospital (OR 1.77, 95% CI 1.23–2.54, *p* = 0.002). The mean difference in length of hospital stay (intervention minus non-intervention) was −0.12 days (95% CI −1.28 to 1.04, *p* = 0.841), indicating no clear difference between groups. Therefore, the unadjusted between-group difference in in-hospital mortality was not supported after overlap-weighted adjustment accounting for covariate differences, whereas a favorable discharge outcome and transfer to a post-acute rehabilitation hospital remained consistently more likely in the intervention period (Table 3).

### 4.7. Sensitivity Analysis Addressing Secular Trends

To assess the influence of secular trends, we conducted a sensitivity analysis restricted to the final 12 months of the non-intervention period and the first 12 months of the intervention period. Unadjusted comparisons revealed no significant difference in in-hospital mortality between the final 12 months of the non-intervention period and the first 12 months of the intervention period (17.0% vs. 15.5%, *p* = 0.288). The favorable discharge outcome rate was higher in the intervention period (79.3% vs. 76.0%, *p* = 0.061); however, this result did not reach statistical significance. The rate of transfer to a post-acute rehabilitation hospital was significantly higher in the intervention period (8.8% vs. 5.6%, *p* = 0.001). Length of hospital stay was 6.0 days in both groups, with no significant between-group difference (*p* = 0.589). In the same subsample, overlap weighting based on the propensity score was applied, and adjusted analyses were performed using robust variances (sandwich estimators). The OR for in-hospital mortality was 1.01 (95% CI 0.61–1.65; overlap-weighted adjusted *p* = 0.983). The likelihood of a favorable discharge outcome was significantly higher in the intervention period (OR 3.69, 95% CI 2.59–5.25; overlap-weighted adjusted *p* < 0.001). The OR for transfer to a post-acute rehabilitation hospital was 1.38 (95% CI 0.83–2.28; overlap-weighted adjusted *p* = 0.216). The mean difference in length of hospital stay (intervention minus non-intervention) was 0.37 days (95% CI −1.10 to 1.85; overlap-weighted adjusted *p* = 0.612) (Supplementary Table S3).

Overall, in the time-restricted analysis, overlap-weighted adjustment continued to show a positive association between the intervention period and a favorable discharge outcome, but no clear between-group differences in in-hospital mortality, rate of transfer to a post-acute rehabilitation hospital, or length of hospital stay.

## 5. Discussion

### 5.1. Study Design and Methodology

This study compared clinical and discharge outcomes between a non-intervention group and an intervention group, defined by implementation of a program designed to initiate acute-phase rehabilitation within 24 h of hospital arrival in a tertiary emergency and critical care center. Given the before–after design, it was not possible to completely eliminate confounding as a result of secular changes, including shifts in case mix and changes in clinical protocols. Moreover, although illness severity was accounted for in the primary adjustment model using the APACHE II score, the possibility of residual confounding stemming from secular changes and concurrent interventions cannot be fully excluded [24]. We applied propensity score overlap weighting to address measured confounding and evaluated covariate balance using SMDs, thereby focusing on inference in the region of greatest clinical comparability between groups [25,26,27]. The post-weighting convergence of covariate balance indicates that the primary analysis achieved substantial adjustment for observed differences; however, the results should still be interpreted as implementation-associated outcomes rather than definitive causal effects of a single therapeutic exposure.

### 5.2. Principal Findings

Two findings of this study merit emphasis. First, although the crude comparison suggested lower in-hospital mortality in the intervention group, this difference disappeared after overlap-weighted adjustment. In contrast, the likelihood of a favorable discharge outcome and the rate of transfer to a rehabilitation hospital remained consistently higher in the intervention group after adjustment. Second, length of stay was longer in the crude comparison but did not differ clearly after adjustment.

To further address concerns regarding secular trends, we conducted a sensitivity analysis restricting the comparison to the most recent 12 months of the non-intervention period and the first 12 months of the intervention period. In that restricted sample, overlap-weighted estimates continued to support a higher likelihood of a favorable discharge outcome in the intervention period, whereas in-hospital mortality, transfer to a rehabilitation hospital, and length of hospital stay did not show clear differences. This pattern underscores the need for cautious interpretation: restricting the time window may alter the case mix and the stability of estimates, and early-phase implementation may have been affected by maturation of the program and contemporaneous changes in ICU procedures. Accordingly, the observed associations should not be framed as definitive evidence of a mortality-reducing intervention, but rather as outcomes associated with a bundled change in practice centered on standardizing readiness and early initiation of rehabilitation.

### 5.3. Interpretation in the Operational Context of an Emergency and Critical Care Center

The findings of this study are plausible when interpreted in the context of a high-turnover emergency and critical care environment. In such settings, the time available to achieve large short-term functional gains during hospitalization is limited, and the principal value of early rehabilitation may lie in preventing rapid deconditioning and promoting early readiness for activity rather than in altering mortality. This aligns with previous research indicating that early mobilization may improve functional outcomes and discharge disposition even when effects on mortality are inconsistent [17,18,21,28,29]. Furthermore, early rehabilitation may operate as a pragmatic “bridge” that strengthens the opportunity for transition to post-acute rehabilitation, which is particularly relevant in Japanese tertiary centers where discharge planning and coordination of transfer are major determinants of care trajectories.

Our data for transfer to a rehabilitation hospital require careful interpretation. They can reflect the potential for recovery and functional needs, but are also influenced by regional bed availability, institutional discharge policies, and family or social factors. Therefore, an increased rate of transfer does not necessarily indicate improved functional status in isolation. However, when accompanied by a higher likelihood of a favorable discharge outcome according to the GOS score, early rehabilitation may be associated with a more favorable status at discharge, although this should not be interpreted as definitive evidence of functional improvement [22].

### 5.4. Mechanistic and Implementation Considerations

A biologically plausible rationale for very early initiation of rehabilitation is grounded in the rapid development of neuromuscular dysfunction and early skeletal muscle wasting during critical illness [1,30,31,32]. Immobility-related muscle loss can occur within days, and neurocritical patients are particularly susceptible because of sedation, mechanical ventilation, and neurological deficits that delay mobilization [30,31,32]. Early rehabilitation may mitigate these processes by promoting activity as soon as physiological stability allows, potentially preserving strength and mobility trajectories.

From an implementation perspective, early rehabilitation is inseparable from analgesia, sedation, and management of delirium and from broader ventilatory care processes [8,9,10,11,33,34,35,36,37]. Protocolized spontaneous awakening and breathing trials, prevention of delirium, and early mobility are synergistic components of the ABCDEF bundle [10], and improvements in this type of integrated care have been associated with better outcomes in multicenter settings [11]. Therefore, the observed associations likely reflect a system-level change in practice rather than the effect of a single isolated therapeutic component.

At the same time, the published evidence indicates that effect sizes of early mobilization and rehabilitation vary substantially according to the context of implementation, severity of illness, and the baseline level of usual care [15,16,17,18,19,20,38,39,40,41,42]. In some trials, more aggressive early mobilization did not improve primary outcomes and raised concerns about feasibility or safety in specific populations [20]. Therefore, our findings should be interpreted within the operational constraints and practice patterns of a tertiary emergency and critical care center, where standardizing early initiation may be the most actionable and scalable aspect of implementation of rehabilitation.

### 5.5. Future Directions

Several directions follow from these findings. First, beyond a binary “intervention versus non-intervention” framing, future work should quantify the dose of rehabilitation (frequency, intensity, mobility milestones achieved) and examine how dose–response relationships vary according to neurological severity and functional status at baseline. Second, interrupted time-series analyses or multicenter designs would strengthen the ability to make causal inferences and improve generalizability. Third, long-term outcomes after discharge, including functional status, quality of life, and need for rehospitalization, should be evaluated to determine whether early implementation of rehabilitation translates into sustained recovery benefits [2,3,4,5,43,44,45,46].

## 6. Limitations

This study has several limitations. First, it had a single-center retrospective historical cohort design, which means that the possibility of residual confounding as a result of secular trends and concurrent interventions cannot be fully excluded. Although overlap weighting improved balance for measured covariates, the study did not systematically capture all key ICU process measures, such as sedation depth, duration of mechanical ventilation, occurrence and severity of delirium, and adherence to other elements of the ABCDEF bundle. Therefore, the specific contribution of the intervention relative to the non-intervention setting cannot be statistically evaluated. Although discharge coordination and major ICU protocols had not materially changed during the study period at our center, residual confounding due to other unmeasured time-dependent factors, including potential staffing changes and the effects of the COVID-19 period, cannot be excluded. Moreover, we did not perform interrupted time-series analyses that explicitly model temporal trends across the transition from the non-intervention group to the intervention group; therefore, any causal interpretation requires caution. Second, because the exposure was a system-level implementation, it was not possible to quantify the content and dose of rehabilitation at the individual level, which limits any mechanistic inference. Third, outcomes were confined to in-hospital and discharge time measures, and long-term outcomes were not assessed. In addition, although Functional Independence Measure data were available after the implementation of that tool, they were not available in the earlier period; therefore, more granular functional assessment could not be compared consistently across periods. Fourth, as with any retrospective analysis of routinely collected data, measurement error and missingness are unavoidable, and results may be sensitive to assumptions regarding the missing-data mechanism despite use of multiple imputation [47,48].

## 7. Conclusions

This study found that implementation of an acute-phase rehabilitation system designed to initiate rehabilitation within 24 h had no clear impact on in-hospital mortality or length of stay in a tertiary emergency and critical care center in Japan. However, patients in whom early rehabilitation was initiated consistently showed a higher likelihood of a favorable discharge outcome and were more likely to be transferred to a rehabilitation hospital. In a high-turnover context where in-hospital functional recovery is structurally constrained, these findings support interpretation of the program as a practical bridge that increases the chances of transition to post-acute rehabilitation rather than as an intervention expected to reduce the short-term risk of mortality.

## Figures and Tables

**Table 1:** Baseline characteristics and early physiological/laboratory variables before overlap weighting.

Variable	Non-Intervention Period	Intervention Period	|SMD|
Cases, n	18,068	2184	
Age (years)	67.0 [47, 79]	68.0 [48, 80]	0.031
Male, n (%)	11,194 (62.0)	1313 (60.1)	0.038
GCS score	14 [6, 15]	12 [3, 15]	0.246
APACHE II score	18 [12, 27]	15 [13, 24]	0.190
Blood pressure (mmHg)			
Systolic	134 [113, 158]	135 [114, 158]	0.082
Diastolic	78 [64, 93]	78 [65, 90]	0.070
Heart rate (bpm)	92 [77, 111]	94 [79, 113]	0.153
Respiratory rate (/min)	21 [18, 27]	22 [18, 30]	0.062
Body temperature (°C)	36.5 [36.0, 37.1]	36.7 [36.2, 37.3]	0.153
pH	7.40 [7.34, 7.46]	7.43 [7.37, 7.49]	0.118
PaCO_2_ (mmHg)	38.2 [33.4, 44.6]	35.8 [31.0, 41.4]	0.165
SaO_2_ (%)	99.2 [96.6, 100]	99.6 [98.0, 100]	0.167
Lactate (mmol/L)	1.8 [1.2, 3.2]	1.7 [1.2, 2.9]	0.291
Na (mmol/L)	139 [137, 141]	139 [137, 141]	0.095
Creatine kinase (U/L)	123 [61, 350]	140 [68, 386]	0.014
AST (U/L)	32 [21, 58]	34 [22, 63]	0.115
ALT (U/L)	24 [15, 41]	25 [15, 43]	0.122
Amylase (U/L)	72 [51, 107]	72 [50, 109]	0.043
BUN (mg/dL)	18.5 [13.7, 26.9]	18.4 [13.6, 27.0]	0.063
Creatinine (mg/dL)	0.86 [0.67, 1.15]	0.85 [0.66, 1.12]	0.038
Albumin (g/dL)	3.7 [3.2, 4.1]	3.7 [3.2, 4.1]	0.214
C-reactive protein (mg/dL)	0.4 [0.09, 2.57]	0.32 [0.08, 2.14]	0.041
D-dimer (mg/L)	3.7 [1.1, 15.6]	1.9 [1.0, 7.9]	0.117

Data are shown as the median [interquartile range] unless otherwise indicated. Abbreviations: APACHE II, Acute Physiology and Chronic Health Evaluation II; ALT, alanine aminotransferase; AST, aspartate aminotransferase; bpm, beats per minute; BUN, blood urea nitrogen; GCS, Glasgow Coma Scale; PaCO_2_, arterial partial pressure of carbon dioxide; SaO_2_, arterial oxygen saturation; SMD, standardized mean difference.

**Table 2:** Unadjusted comparisons of outcomes between groups.

Variable	Non-Intervention	Intervention	*p*-Value
Cases, n	18,068	2184	
In-hospital mortality n (%)	3650 (20.2)	306 (14.0)	<0.001
Favorable outcome n (%)	12,250 (67.8)	1782 (81.6)	<0.001
Rehabilitation transfer n (%)	1102 (6.1)	269 (12.3)	<0.001
Length of stay (days)	6.0 [2–16]	7.0 [2–16]	<0.001

Data are shown as the number (percentage) or the median [interquartile range] as appropriate. A favorable outcome was defined as a Glasgow Outcome Scale score indicating good recovery or moderate disability at discharge.

**Table 3:** Overlap-weighted adjusted associations between implementation of the rehabilitation system and outcomes.

Variable	Effect Measure	Estimate	95% CI	*p*-Value
In-hospital mortality	OR	1.17	0.81–1.69	0.396
Favorable outcome	OR	1.74	1.29–2.36	<0.001
Rehabilitation transfer	OR	1.77	1.23–2.54	0.002
Length of stay (days)	Mean diff.	−0.12	–1.28, 1.04	0.841

Effect estimates were obtained using overlap weighting with robust (sandwich) standard errors. Abbreviations: CI, confidence interval; Mean diff., mean difference (intervention minus non-intervention); OR, odds ratio (intervention vs. non-intervention); OW, overlap weighting.

## Data Availability

The data presented in this study are available on request from the corresponding author due to ethical restrictions.

## Supplementary Materials

The following supporting information can be downloaded at: https://www.mdpi.com/article/10.3390/life16040587/s1, Table S1. Diagnostic categories at presentation by study group (non-intervention vs. intervention). Table S2. Standardized mean differences before and after overlap weighting. Table S3. Overlap-weighted adjusted associations in the restricted 12-month sensitivity analysis.

## References

[1] Vanhorebeek I., Latronico N., Van den Berghe G. (2020). ICU-acquired weakness. Intensive Care Med..

[2] Needham D.M., Davidson J., Cohen H., Hopkins R.O., Weinert C., Wunsch H., Zawistowski C., Bemis-Dougherty A., Berney S.C., Bienvenu O.J. (2012). Improving long-term outcomes after discharge from intensive care unit: Report from a stakeholders’ conference. Crit. Care Med..

[3] Herridge M.S., Tansey C.M., Matte A., Tomlinson G., Diaz-Granados N., Cooper A., Guest C.B., Mazer C.D., Mehta S., Stewart T.E. (2011). Functional disability 5 years after acute respiratory distress syndrome. N. Engl. J. Med..

[4] Pandharipande P.P., Girard T.D., Jackson J.C., Morandi A., Thompson J., Pun B., Brummel N., Hughes C., Vasilevskis E., Shintani A. (2013). Long-term cognitive impairment after critical illness. N. Engl. J. Med..

[5] Rengel K.F., Hayhurst C.J., Pandharipande P.P., Hughes C.G. (2019). Long-term cognitive and functional impairments after critical illness. Anesth. Analg..

[6] Goldberg T.E., Chen C., Wang Y., Jung E., Swanson A., Ing C., Garcia P.S., Whittington R.A., Moitra V. (2020). Association of delirium with long-term cognitive decline: A meta-analysis. JAMA Neurol..

[7] Ely E.W., Margolin R., Francis J., May L.R., Truman B.R., Dittus R., Speroff T., Gautam S., Bernard G.R., Inouye S.K. (2001). Evaluation of delirium in critically ill patients: Validation of the Confusion Assessment Method for the Intensive Care Unit (CAM-ICU). Crit. Care Med..

[8] Barr J., Fraser G.L., Puntillo K., Ely E.W., Gélinas C., Dasta J.F., Davidson J.E., Devlin J.W., Kress J.P., Joffe A.M. (2013). Clinical practice guidelines for the management of pain, agitation, and delirium in adult patients in the intensive care unit. Crit. Care Med..

[9] Devlin J.W., Skrobik Y., Gélinas C., Needham D.M., Slooter A.J.C., Pandharipande P.P., Watson P.L., Weinhouse G.L., Nunnally M.E., Rochwerg B. (2018). Clinical practice guidelines for the prevention and management of pain, agitation/sedation, delirium, immobility, and sleep disruption in adult patients in the ICU. Crit. Care Med..

[10] Ely E.W. (2017). The ABCDEF bundle: Science and philosophy of how ICU liberation serves patients and families. Crit. Care Med..

[11] Pun B.T., Balas M.C., Barnes-Daly M.A., Thompson J.L., Aldrich J.M., Barr J., Byrum D., Carson S.S., Devlin J.W., Engel H.J. (2019). Caring for critically ill patients with the ABCDEF bundle: Results of the ICU Liberation Collaborative in over 15,000 adults. Crit. Care Med..

[12] Bailey P., Thomsen G.E., Spuhler V.J., Blair R., Jewkes J., Bezdjian L., Veale K., Rodriquez L., Hopkins R.O. (2007). Early activity is feasible and safe in respiratory failure patients. Crit. Care Med..

[13] Morris P.E., Goad A., Thompson C., Taylor K., Harry B., Passmore L., Ross A., Anderson L., Baker S., Sanchez M. (2008). Early intensive care unit mobility therapy in the treatment of acute respiratory failure. Crit. Care Med..

[14] Onda H., Yokobori S. (2025). Early rehabilitation and improved outcomes in patients with status epilepticus: Evidence from cases presenting to the emergency department. Epilepsia Open.

[15] Hodgson C.L., Bailey M., Bellomo R., Berney S., Buhr H., Denehy L., Gabbe B., Harrold M., Higgins A., Iwashyna T.J. (2016). A binational multicenter pilot feasibility randomized controlled trial of early goal-directed mobilization in the ICU. Crit. Care Med..

[16] Doiron K.A., Hoffmann T.C., Beller E.M. (2018). Early intervention (mobilization or active exercise) for critically ill adults in the intensive care unit. Cochrane Database Syst. Rev..

[17] Zhang L., Hu W., Cai Z., Liu J., Wu J., Deng Y., Yu K., Chen X., Zhu L., Ma J. (2019). Early mobilization of critically ill patients in the intensive care unit: A systematic review and meta-analysis. PLoS ONE.

[18] Wang J., Ren D., Liu Y., Wang Y., Zhang B., Xiao Q. (2020). Effects of early mobilization on the prognosis of critically ill patients: A systematic review and meta-analysis. Int. J. Nurs. Stud..

[19] Menges D., Seiler B., Tomonaga Y., Schwenkglenks M., Puhan M.A., Yebyo H.G. (2021). Systematic early versus late mobilization or standard early mobilization in mechanically ventilated adult ICU patients: A systematic review and meta-analysis. Crit. Care.

[20] (2022). TEAM Study Investigators; ANZICS Clinical Trials Group. Early active mobilization during mechanical ventilation in the ICU. N. Engl. J. Med..

[21] Schweickert W.D., Pohlman M.C., Pohlman A.S., Nigos C., Pawlik A.J., Esbrook C.L., Spears L., Miller M., Franczyk M., Deprizio D. (2009). Early physical and occupational therapy in mechanically ventilated, critically ill patients: A randomised controlled trial. Lancet.

[22] Jennett B., Bond M. (1975). Assessment of outcome after severe brain damage. Lancet.

[23] Knaus W.A., Draper E.A., Wagner D.P., Zimmerman J.E. (1985). APACHE II: A severity of disease classification system. Crit. Care Med..

[24] Penfold R.B., Zhang F. (2013). Use of interrupted time series analysis in evaluating health care quality improvements. Acad. Pediatr..

[25] Li F., Thomas L.E., Li F. (2019). Addressing extreme propensity scores via the overlap weights. Am. J. Epidemiol..

[26] Thomas L.E., Li F., Pencina M.J. (2020). Overlap weighting: A propensity score method that mimics attributes of a randomized clinical trial. JAMA.

[27] Austin P.C. (2009). Balance diagnostics for comparing the distribution of baseline covariates between treatment groups in propensity-score matched samples. Stat. Med..

[28] Tipping C.J., Harrold M., Holland A., Romero L., Nisbet T., Hodgson C.L. (2017). The effects of active mobilisation and rehabilitation in ICU on mortality and function: A systematic review. Intensive Care Med..

[29] Schaller S.J., Anstey M., Blobner M., Edrich T., Grabitz S.D., Gradwohl-Matis I., Heim M., Houle T., Kurth T., Latronico N. (2016). Early, goal-directed mobilisation in the surgical intensive care unit: A randomised controlled trial. Lancet.

[30] De Jonghe B., Sharshar T., Lefaucheur J.P., Authier F.-J., Durand-Zaleski I., Boussarsar M., Cerf C., Renaud E., Mesrati F., Carlet J. (2002). Paresis acquired in the intensive care unit: A prospective multicenter study. JAMA.

[31] Puthucheary Z.A., Rawal J., McPhail M., Connolly B., Ratnayake G., Chan P., Hopkinson N.S., Padhke R., Dew T., Sidhu P.S. (2013). Acute skeletal muscle wasting in critical illness. JAMA.

[32] Kumar M.A., Romero F.G., Dharaneeswaran K. (2020). Early mobilization in neurocritical care patients. Curr. Opin. Crit. Care.

[33] Kress J.P., Pohlman A.S., O’Connor M.F., Hall J.B. (2000). Daily interruption of sedative infusions in critically ill patients undergoing mechanical ventilation. N. Engl. J. Med..

[34] Girard T.D., Kress J.P., Fuchs B.D., Thomason J.W., Schweickert W.D., Pun B.T., Taichman D.B., Dunn J.G., Pohlman A.S., Kinniry P.A. (2008). Efficacy and safety of a paired sedation and ventilator weaning protocol for mechanically ventilated patients in intensive care (Awakening and Breathing Controlled trial): A randomised controlled trial. Lancet.

[35] Pandharipande P.P., Pun B.T., Herr D.L., Maze M., Girard T.D., Miller R.R., Shintani A.K., Thompson J.L., Jackson J.C., Deppen S.A. (2007). Effect of sedation with dexmedetomidine vs lorazepam on acute brain dysfunction in mechanically ventilated patients: The MENDS randomized controlled trial. JAMA.

[36] Riker R.R., Shehabi Y., Bokesch P.M., Ceraso D., Wisemandle W., Koura F., Whitten P., Margolis B.D., Byrne D.W., Ely E.W. (2009). Dexmedetomidine vs midazolam for sedation of critically ill patients: A randomized trial. JAMA.

[37] Jakob S.M., Ruokonen E., Grounds R.M., Sarapohja T., Garratt C., Pocock S.J., Bratty J.R., Takala J. (2012). Dexmedetomidine vs midazolam or propofol for sedation during prolonged mechanical ventilation: Two randomized controlled trials. JAMA.

[38] Kayambu G., Boots R., Paratz J. (2013). Physical therapy for the critically ill in the ICU: A systematic review and meta-analysis. Crit. Care Med..

[39] Morris P.E., Berry M.J., Files D.C., Thompson J.C., Hauser J., Flores L., Dhar S., Chmelo E., Lovato J., Case L.D. (2016). Standardized rehabilitation and hospital length of stay among patients with acute respiratory failure: A randomized clinical trial. JAMA.

[40] Harrold M.E., Salisbury L.G., Webb S.A., Allison G.T. (2015). Early mobilisation in intensive care units in Australia and Scotland: A prospective, observational cohort study examining mobilisation practises and barriers. Crit. Care.

[41] Dubb R., Nydahl P., Hermes C., Schwabbauer N., Toonstra A., Parker A.M., Kaltwasser A., Needham D.M. (2016). Barriers and strategies for early mobilization of patients in intensive care units. Ann. Am. Thorac. Soc..

[42] Nydahl P., Sricharoenchai T., Chandra S., Kundt F.S., Huang M., Fischill M., Needham D.M. (2017). Safety of patient mobilization and rehabilitation in the intensive care unit: Systematic review with meta-analysis. Ann. Am. Thorac. Soc..

[43] Hua M., Gong M.N., Brady J., Wunsch H. (2015). Early and late unplanned rehospitalizations for survivors of critical illness. Crit. Care Med..

[44] Hill A.D., Fowler R.A., Pinto R., Herridge M.S., Cuthbertson B.H., Scales D.C. (2016). Long-term outcomes and healthcare utilization following critical illness: A population-based study. Crit. Care.

[45] Griffiths J., Hatch R.A., Bishop J., Morgan K., Jenkinson C., Cuthbertson B.H., Brett S.J. (2013). An exploration of social and economic outcome and associated health-related quality of life after critical illness in general intensive care unit survivors: A 12-month follow-up study. Crit. Care.

[46] Jackson J.C., Ely E.W., Morey M.C., Anderson V.M.M., Denne L.B.M., Clune J., Siebert C.S., Archer K.R., Torres R., Janz D. (2012). Cognitive and physical rehabilitation of intensive care unit survivors: Results of the RETURN randomized controlled pilot investigation. Crit. Care Med..

[47] Sterne J.A.C., White I.R., Carlin J.B., Spratt M., Royston P., Kenward M.G., Wood A.M., Carpenter J.R. (2009). Multiple imputation for missing data in epidemiological and clinical research: Potential and pitfalls. BMJ.

[48] White I.R., Royston P., Wood A.M. (2011). Multiple imputation using chained equations: Issues and guidance for practice. Stat. Med..

