# Supplementary material for: Implementation and Outcomes of an Early Rehabilitation Program in a Tertiary Emergency and Critical Care Center: A Single-Center Historical Cohort Study
Source: Life (Basel). 2026 Apr 1;16(4):587. doi: 10.3390/life16040587 (PMC13117265; doi:10.3390/life16040587)
Supplement: Supplementary file 1 [file life-16-00587-s001.zip › life-4206913-supplementary.pdf]

Supplementary materials

Table S1. Diagnostic categories at presentation by study group (non-intervention vs intervention)

Parameter	Non-intervention group	Intervention group	|SMD|
Cases, n	18,068	2,184	
Sepsis (%)	357 (2.0%)	54 (2.5%)	0.034
Trauma (%)	2,568 (14.2%)	291 (13.3%)	0.026
Respiratory disease (%)	1,473 (8.2%)	332 (15.2%)	0.221
Cardiovascular disease (%)	2,232 (12.4%)	238 (10.9%)	0.045
Gastrointestinal disease (%)	1,957 (10.8%)	226 (10.3%)	0.016
Stroke (%)	833 (4.6%)	56 (2.6%)	0.110
Poisoning (%)	1,272 (7.0%)	197 (9.0%)	0.073
Other (%)	7,376 (40.8%)	790 (36.2%)	0.096

Table S2. Standardized mean differences before and after overlap weighting

Variable	Absolute SMD before OW	Absolute SMD after OW
Cases, n	18,068	2,184
Age (years)	0.031	0.001
Male, n (%)	0.038	0.000
GCS score	0.246	0.001
APACHE II score	0.190	0.001
Blood pressure (mmHg)		
Systolic	0.082	0.002
Diastolic	0.070	0.002
Heart rate (bpm)	0.153	0.002
Respiratory rate (/min)	0.062	0.001
Body temperature (°C)	0.153	0.001
pH	0.118	0.006
$PaCO_2$ (mmHg)	0.165	0.001
SaO_2 (%)	0.167	0.002
Lactate (mmol/L)	0.291	0.000
Na (mmol/L)	0.095	0.004

Creatine kinase (U/L)	0.014	0.000
AST (U/L)	0.115	0.000
ALT (U/L)	0.122	0.000
Amylase (U/L)	0.043	0.000
BUN (mg/dL)	0.063	0.000
Creatinine (mg/dL)	0.038	0.000
Albumin (g/dL)	0.214	0.001
C-reactive protein (mg/dL)	0.041	0.000
D-dimer (mg/L)	0.117	0.000

Abbreviations: APACHE II, Acute Physiology and Chronic Health Evaluation II; ALT, alanine aminotransferase; AST, aspartate aminotransferase; bpm, beats per minute; BUN, blood urea nitrogen; GCS, Glasgow Coma Scale; OW, overlap weighting; $PaCO_2$, arterial partial pressure of carbon dioxide; SaO_2, arterial oxygen saturation; SMD, standardized mean difference

Table S3. Overlap-weighted adjusted associations in the restricted 12-month sensitivity analysis

Parameter	Unadjusted P-value	Effect measure	Estimate	95% CI	OW-adjusted P-value
In-hospital mortality	0.288	OR	1.01	0.61–1.65	0.983
Favorable outcome	0.061	OR	3.69	2.59–5.25	<0.001
Rehabilitation transfer	0.001	OR	1.38	0.83–2.28	0.216
Length of stay (days)	0.589	Mean diff.	0.37	−1.10, 1.85	0.612

Effect estimates were obtained using overlap weighting with robust (sandwich) standard errors.

Abbreviations: CI, confidence interval; Mean diff., mean difference (intervention minus non-intervention); OR, odds ratio; OW, overlap weighting
